# Updated Systematic Review on the Role of Brain Invasion in Intracranial Meningiomas: What, When, Why?

**DOI:** 10.3390/cancers14174163

**Published:** 2022-08-27

**Authors:** Lara Brunasso, Lapo Bonosi, Roberta Costanzo, Felice Buscemi, Giuseppe Roberto Giammalva, Gianluca Ferini, Vito Valenti, Anna Viola, Giuseppe Emmanuele Umana, Rosa Maria Gerardi, Carmelo Lucio Sturiale, Alessio Albanese, Domenico Gerardo Iacopino, Rosario Maugeri

**Affiliations:** 1 Neurosurgical Clinic AOUP “Paolo Giaccone”, Post Graduate Residency Program in Neurologic Surgery, Department of Biomedicine Neurosciences and Advanced Diagnostics, School of Medicine, University of Palermo, 90127 Palermo, Italy; 2 Department of Radiation Oncology, REM Radioterapia SRL, 95125 Catania, Italy; 3 Gamma Knife Center, Trauma Center, Department of Neurosurgery, Cannizzaro Hospital, 95100 Catania, Italy; 4 Division of Neurosurgery, Fondazione Policlinico Universitario A. Gemelli IRCCS, Università Cattolica del Sacro Cuore, 00100 Rome, Italy

**Keywords:** meningioma, intracranial meningioma, meningioma management, brain tumor invasive growth, brain invasion, brain edema, meningioma prognosis

## Abstract

**Simple Summary:**

Meningioma is still the most common adult tumor of the CNS, most of which are slow-growing, benign tumors and could even be accidentally diagnosed; nonetheless, they sometimes show more aggressive behavior with higher recurrence rates and relatively reduced overall survival. Assuming this, in recent years, scientific research has been accelerated, looking for new insights and applications that could improve preoperative investigation, tailor surgical planning, and strongly impact meningioma patients’ prognosis. Many fields have been developed, and the detection of brain invasion has firmly gained its potential role, leading to the revised version of WHO for CNS tumors in 2016 as a further criterion for defining atypia. Further studies are still ongoing to assess a widely accepted application of BI evaluation in intracranial meningioma management.

**Abstract:**

Several recent studies are providing increasing insights into reliable markers to improve the diagnostic and prognostic assessment of meningioma patients. The evidence of brain invasion (BI) signs and its associated variables has been focused on, and currently, scientific research is investing in the study of key aspects, different methods, and approaches to recognize and evaluate BI. This paradigm shift may have significant repercussions for the diagnostic, prognostic, and therapeutic approach to higher-grade meningioma, as long as the evidence of BI may influence patients’ prognosis and inclusion in clinical trials and indirectly impact adjuvant therapy. We intended to review the current knowledge about the impact of BI in meningioma in the most updated literature and explore the most recent implications on both clinical practice and trials and future directions. According to the PRISMA guidelines, systematic research in the most updated platform was performed in order to provide a complete overview of characteristics, preoperative applications, and potential implications of BI in meningiomas. Nineteen articles were included in the present paper and analyzed according to specific research areas. The detection of brain invasion could represent a crucial factor in meningioma patients’ management, and research is flourishing and promising.

## 1. Introduction

In recent years, the advent of new technologies and their consequential potential prognostic impact on patients has laid the groundwork for a radical change in the neurosurgical and oncological approach to intracranial meningioma management. Surgical excision and/or postoperative radiotherapy have always been the mainstay of the treatment of intracranial meningioma; currently, the management of this CNS tumor is rapidly evolving to a more complex algorithm of factors to be inspected carefully. Tumor-immune infiltrate, tumor-immune cell interactions, immunogenomics, immunotherapy, radiological preoperative investigation through latest generation software, and radiomics are some of the major growing fields in meningioma research, and the concept of brain invasion (BI) has developed, becoming an independent criterion for the diagnosis of atypia, even in Grade I (WHO 2016) meningiomas [1]. According to the WHO classification, Grade I meningiomas are usually related to a better outcome, with a very low percentage of tumor recurrence and, when compatible with tumor characteristics treated with surgery alone, no need for follow-up; nevertheless, several cases reported in the literature show a mismatch in tumor recurrence for Grade I meningiomas, and after few years of scientific investigations, BI was finally accepted and added in the revised version of WHO for CNS tumors in 2016 as a further criterion of atypia [2]. All this has led to a deep revolution, still in progress, in meningioma tumor management, where the use of radiomics parameters and radiological preoperative planning, molecular markers, microscopical detection of BI, and other features may lead to an ever-growing tailored approach to the patient and a more favorable prognosis [3,4].

The aim of this paper is to provide a perspective of the current data pertaining to BI investigations and acknowledgments in intracranial meningiomas, their results achieved in terms of diagnosis and prognosis, and to identify potential tumor features that may affect medical and surgical decision-making and treatment outcomes. The authors give a panoramic view of all BI parameters currently used and their impact on daily practice, identifying limitations and gaps, as well as accuracy grades and promising future directions in meningioma management. Lastly, this review aims to expand and, at the same time, gather published studies focusing on all the available tools to detect BI and to improve patients’ prognostic stratification.

## 2. Materials and Methods

**Figure 1:**
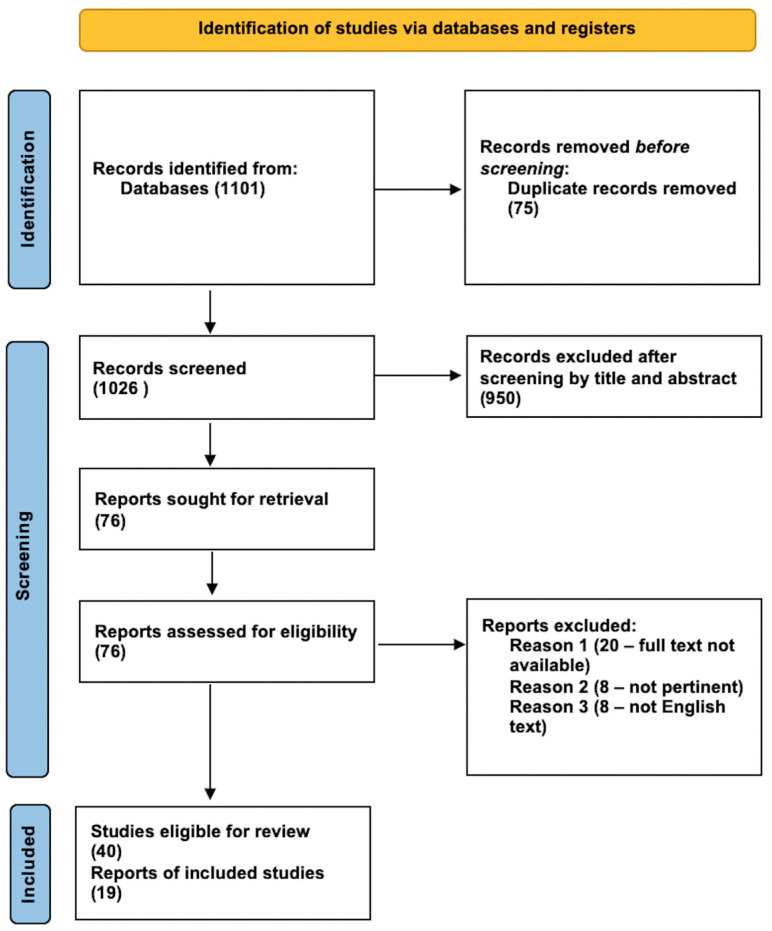
Flow diagram of the results of this systematic review according to PRISMA guidelines.

Following the Preferred Reporting Items for Systematic Reviews and Meta-Analyses (PRISMA) statement, a literature review on the characteristics and the role of BI evidence in meningioma was systematically conducted. This review was not recorded on prospective registers; thus, review protocol was not prospectively available. PubMed, Web of Science, the Cochrane Library, and Scopus databases were searched from database inception to May 2022 using the following search string: “Brain invasion AND meningioma”, “Brain invasion AND meningioma AND MRI sign”, “Peritumoral brain oedema AND meningioma AND brain invasion”, “Meningioma” AND “proteomics”, “Meningioma” AND “proteomics” AND “cerebrospinal fluid” OR “CSF”. No restrictions on the year of publication or type of paper were made. Three authors (L.Br, L.Bo., F.B.) independently screened abstracts for eligibility. The literature search provided 1101 results, and we excluded all duplicates (n = 75), off-topic titles and/or abstracts (n = 958), papers with no available full-text (n = 20), and languages other than English (n = 8). Finally, 40 papers were eligible for this review, and 19 papers were considered consistent with the focus of the present paper and included and analyzed. The following features were extracted: number of patients, sex, age, tumor location, preoperative edema volume identified through the use of MRI (Magnetic Resonance Imaging), whenever mentioned, WHO grade, histologic subtypes, eventual adjuvant therapies, radiological and/or histological presence of brain invasion, and mitotic index (Figure 1).

## 3. Results

**Figure 2:**
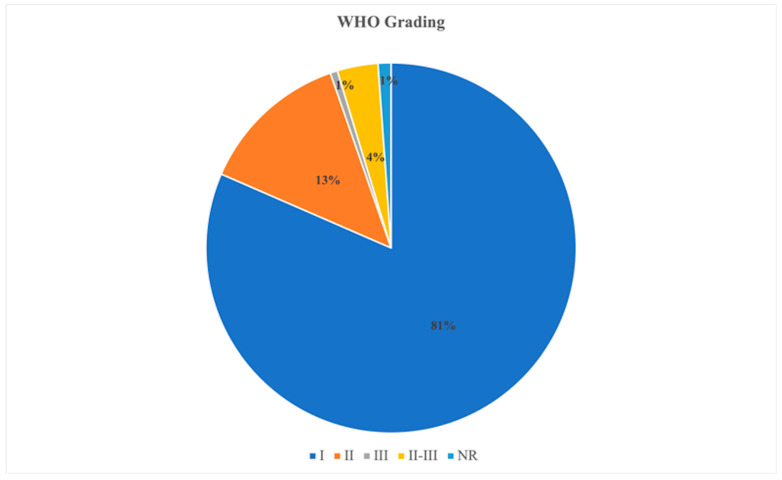
Pie-chart of the most common histological grades distribution for meningiomas included in the papers selected for the present systematic review.

**Figure 3:**
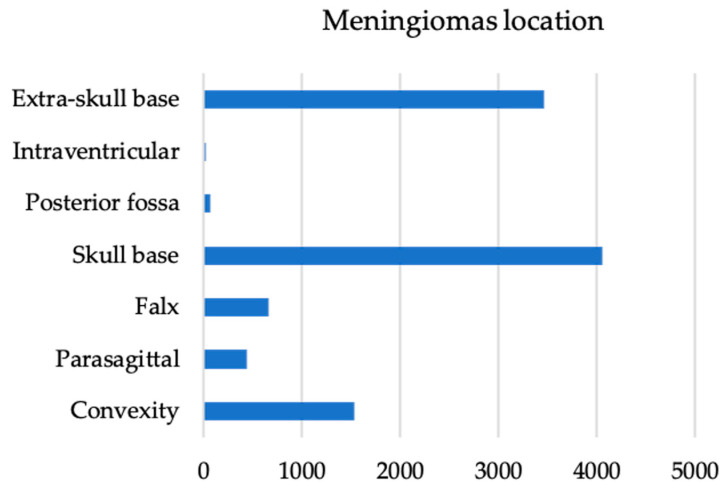
Diagram of the most frequent locations of meningiomas included in the papers selected for the present systematic review.

Nineteen published studies that met the above-mentioned inclusion criteria were included in this review. A qualitative analysis (Table 1, Figure 2 and Figure 3) was performed on 19 papers for a total of 10,218 patients, including meningioma of various grades, 2978 males (29.15%) and 7220 females (70.66%); in 20 patients, the sex was not specified (0.19%). The mean age was 60.08 ± 4.31 years. The most frequent localization was in the skull base (4055), followed by an extra-skull base (unspecified) (3459), convexity (1531), falx (663), parasagittal (433), posterior cranial fossa (63), and intraventricular (14). In addition, meningioma grade according to the World Health Organization (WHO) was recorded: Grade I in 8331 patients, Grade II in 1343 patients, Grade III in 65 patients, and Grades II–III in 363 patients. Grade not reported in 116 patients. The presence of brain invasion was reported in 1731 patients, while it was absent in 6090 patients. A high mitotic index was reported only in 8/19 papers [3,5,6,7,8,9,10,11,12,13,14,15,16,17,18,19] and a low mitotic index only in 4/19 papers [2,12,15,19]. A preoperative MRI analysis in terms of arachnoid layer alteration, irregular tumor shape, eventual calcifications, capsular contrast enhancement, and heterogeneous enhancement, as well as fractal parameters analysis, was carried out only in 4/19 papers [8,9,11,13]. In addition, a preoperative edema volume analysis was reported in 6/19 papers with a mean volume of 129.6 ± 115 cm^3^, particularly in skull-base invasive meningiomas [6,9,11,14]. Patients’ demographics are shown in Table 1.

## 4. Discussion

### 4.1. Meningioma “Brain Invasion” Concept Evolution

In recent years, a set of definitions and assessment methods for BI have been described in the scientific literature [23,24]. Initially described as tumor cells growing along the Virchow–Robin spaces [18], the most recent definition of BI consists in detecting tumor cells infiltrating the adjacent brain parenchyma without interposing a connective tissue layer (leptomeninges) and a well-defined separating plane [6], together with possible reactive astrocytosis in surrounding brain parenchyma [25].

**Figure 4:**
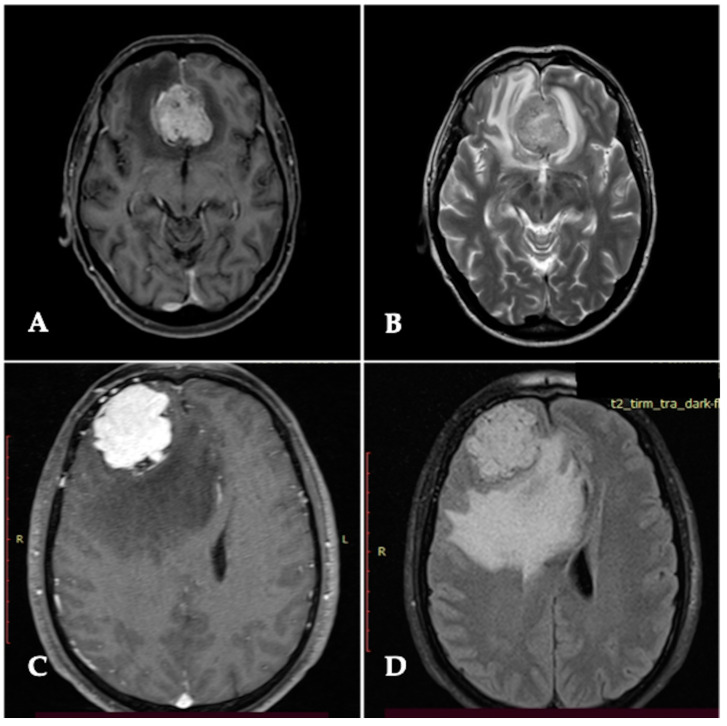
T2-weighted and FLAIR MRI demonstrating the degree of peritumoral edema between a skull-base meningioma (**A**,**B**) and a convexity meningioma (**C**,**D**), respectively. Some authors correlate it to tumor grade, consistent with lower grade meningioma in skull-base located meningioma and higher grade in convexity located meningioma.

Correct tumor sampling during surgery and the adequate combination between histopathological and intraoperative assessment of CNS invasion definitely assess the real presence of BI and achieve real prognostic significance. Behling et al. [13] shrewdly analyzed a series of crucial aspects in this regard. Apart from ethical aspects of intraoperative histological sampling on the border between tumor and brain parenchyma, at times, surgical specimens collected to assess invasion lacked an effective infiltrative interface [26]. Several factors could also potentially influence intraoperative tumor sampling: first, tumor location as with skull-base meningioma, where the sampling may result risky and hazardous; second, the proximity of tumor to brain eloquent areas, where even the complete surgical resection may be technically demanding; third, the choice of surgical approach may lead to “maneuvering” difficulties. Some authors have argued that the use of cavitron ultrasonic surgical aspirators (CUSAs), commonly used in neurosurgical practice, with subsequent tissue loss may further contribute to the difficulty in selective sampling of interface meningioma tissue, leading also to an underestimation of tumor grading and consequentially prognostic and therapeutic implications [1]. The morphological pattern of BI is also variable. To determine BI, meningioma cells need to adhere to resident cells or to the extracellular matrix (ECM) to migrate into the intracellular space. Some of the main inherent studies were based on Grade I WHO tumors in order to reduce the potential influence of the infiltrative behavior of a higher grade, where aggressive cancer growth tends to be associated with the disruption of the pial–glial basement membrane (BM) [27]. Three major patterns of infiltration of brain parenchyma were described in the scientific literature as peritumoral edema (PTBE): tongue- or finger-like, where protrusion of tumor spread through the adjacent brain parenchyma, which is the most frequently reported; diffuse growth, where single cells are supposed to spread into the adjacent brain parenchyma; and clustered “nests”, defined also as islands of tumors cells [24,25,27]. Different biological behavior is associated with tumor location and convexity, and meningiomas are documented more aggressive compared with skull-base meningiomas (Figure 4). A gender-related basis was described, such that BI is most frequently encountered in males, but also the morphological pattern of invasion differs for sex; it was reported that males show finger-like invasions in most cases, and females mainly show clustered-type invasion [15,20]. An age-related basis was also considered, such as brain-invasive meningioma being common in older patients [15]. However, further studies are needed to verify this hypothesis. Interestingly, BI was significantly most frequently associated with non-skull-base meningiomas [28], regardless of histological grade, suggesting a possible role of location in aggressive tumor behavior.

Although not listed as a grading feature for more than 2 decades, since 1993, meningiomas showing BI demonstrated a worse prognosis than other meningiomas with no sign of BI [24]. In this context, the definition and the prognostic role of BI have become a contentious issue in the scientific literature [2], and there have been conflicting data from various studies [3,17,20,29]. Until the 2007 WHO Classification, BI in meningioma did not represent a per se criterion of atypia but was only proposed as a progression risk factor [30]. Given the high amount of evidence of increased risk of tumor progression in the presence of BI in meningioma, the 2016 edition of the WHO Classification of CNS tumors cataloged the evidence of BI as a standalone criterion for the diagnosis of an atypical Grade II meningioma [24]. Microscopic evidence of BI, even in the absence of further histopathological criteria of atypia or in the absence of mitotic activity, achieved the highest clinical relevance and was considered sufficient to directly impact tumor grading as atypical [31]. The last edition (5th) of the WHO Classification of CNS tumors, published in 2021, has classified meningiomas into a single type with 15 different morphological-related subtypes [23], and the presence of BI still remains a criterion for considering atypia in meningiomas [31].

### 4.2. Recognition of Brain Invasion: The Role of Technologies

Few preoperative diagnostic methods are available that could help in suspecting the meningioma grading, while histological examination is still crucial to characterize all CSN tumors. Some clinical symptoms could show indirectly microscopically detected BI. For example, a history of preoperative seizures is related to a high rate of histologically brain-invasive meningioma, and this is an independent factor of patients’ age, sex, WHO grade, and remarkably tumor location peritumoral edema or tumor volume [8,9]. In addition, preoperative behavior changes in patients with meningiomas were associated with the presence of BI at postoperative histological examination [7,24].

#### 4.2.1. MRI Findings

Diagnostic imaging is a primary building block in the diagnostic algorithm of a CNS tumor worldwide. MRI efficiently defines morphological features of meningiomas and is actually the gold standard for preoperative imaging. Many studies have investigated an MRI-shape analysis, with the association between shape and meningioma aggressiveness [32]. Friconnet et al. [21] examined different parameters in supratentorial meningioma shape such as circularity, solidity, and fractal dimension (FD); in particular, they related low circularity with histological brain invasion (*p* = 0.0016) and high NSB, RLS, FD, and low solidity with histological brain invasion (respectively, *p* = 0.0027, *p* = 0.00194, *p* = 0.0079, and *p* = 0.00038).

Other works have investigated how preoperative radiological classification could support histopathological grading and prognostic evaluation with reasonable accuracy [11,19,33]. Interestingly, a score was described by Friconnet et al. considering incomplete CSF rims, size, peritumoral flow voids, and skull-base meningioma to predict pial vascularization of supratentorial intracranial meningioma, and a higher rate of BI was found when the score was >6 [11]. In their retrospective analysis, Liu and colleagues employed different radiological parameters such as hyperintensity on diffusion-weighted MRI (DWI), heterogeneity on T1-weighted gadolinium, enhanced MRI, disruption of arachnoid at the brain tumor interface, and peritumoral edema (PTE) on T2-weighted MRI and tumor shape, to build a scoring system related to overall survival (OS).

Another retrospective study conducted by Ong et al. [34] showed various MRI (and also MRA or TCA in some cases) features that could be associated with BI in meningioma, such as edema volume, enlarged pial feeding arteries, and the presence of the cerebral spinal fluid (SF) cleft. In particular, edema volume was significantly and statistically related in the brain-invasive meningioma group compared to the non-brain-invasive group (*p* = 0.02), and the presence of a complete CSF cleft was found in 0% of brain-invasive meningiomas. This result was confirmed by other authors [35]. In addition, Adeli et al. [8] affirmed that the cut-off point for edema volume could be represented at 3.64 cm as a discrimination threshold for BI (AUC = 0.718).

It should also be remembered that Grade I meningioma frequently shows peritumoral brain edema without BI. This could be attributed to various proposed etiologies: compressive ischemia with compromise of the blood–brain barrier (BBB), vascular shunting due to paralysis of pial microvessels, mechanical venous obstruction, elevated hydrostatic pressure within the tumor, and secretory–excretory phenomena. Thus, while atypical and malignant meningiomas can cause peritumoral edema by invading the brain, in some cases, peritumoral edema may not correlate with the presence of BI with clearly prognostic consequences [35]. However, the outcomes of these imaging signs, defined as “traditional semantics findings”, have not been widely validated [35].

#### 4.2.2. Radiomics

With the spread of the BI concept and its potential prognostic value, the first radiological studies concerning BI evaluation employed a qualitative approach and revealed current limitations in medical imaging techniques and human subjective interpretation, together with the demanding expectations in tumor treatment and prognosis and the continuous demand for flexibility and speed of response guaranteed from advanced technologies. This context provided the opportunity for the development and integration of more advanced artificial intelligence (AI) methodologies and their subvisual feature analysis as radiomics. Radiomics is a machine-learning (ML) methodology that allows extraction of quantitative and reproducible tissue and lesion features from diagnostic images, called radiomics features [36]. It represents a new, low-cost, reliable, and promising tool in the individualized oncological management of meningioma patients [37,38] and provides some advantages compared to the previous qualitative radiological interpretations; in fact, by using defined algorithms, radiomics analysis could capture and reveal more specific information of the disease undetectable for the human eye and provide analysis about intensity distributions, spatial relationships, and texture heterogeneity within a region, as well as across the entire volume of the tumor [37,38,39,40], identifying invisible different subregions, which is not possible through biopsies, and analyzing their potential changes over time on serial imaging [41,42,43].

For this reason. different works have been shared in the most updated literature about personal experience in radiomics analysis with different software using and algorithm creation. Joo and colleagues [14] constructed, for example, a combined model of six radiomics features from the brain-to-tumor interface on T2WI and CE-T1WI and the volume of peritumoral edema, and it showed a better prediction of BI and marked improved diagnostic value over the edema/volume-only model. Similar results were shared by other working groups [44,45,46,47], underlining the great potential of radiomics in this particular subfield and the consequent decision-making implications.

Radiomics features could, alone or in combination with other developing fields (genomic, transcriptomic, or proteomic data) or with demographic and histologic data, help in the diagnostic and treatment of tumor challenges and in patient stratification [37,38].

#### 4.2.3. Proteomics on CSF

In recent years, great interest has been directed toward proteomics and its potential diagnostic, prognostic, and therapeutic implications. Proteomics refers to the study of proteins in biological systems, and the great strength is that it could provide direct information about the structural, signaling, and enzymatic building blocks of the human body [48]. This method aims to characterize the entire “protein kit” contained in a cell at a given time and to identify from body fluids analysis possible biomarkers of great functional importance; in some cases, these proteins can be represented by products secreted by pathological cells, affected by a disease. In the neuro-oncological field, proteogenomic characterization has reinforced the robustness of the newly defined molecular groups of brain tumors and uncovered highly abundant and group-specific protein targets in meningioma [49,50,51,52].

CSF has been recently identified as a reliable source of protein biomarkers for brain tumors for its high protein content and its direct contact with the brain. Some authors have shared their experience in proteome analysis of CSF. Kim and colleagues [53] found that there was a difference between CSF composition of patients with meningioma and patient-control population in eleven different protein expression: serum albumin precursor, apolipoprotein E (Apo E), apolipoprotein J precursor (Apo J), transthyretin precursor (TTR), prostaglandin D2 synthase (PTGDS), proapolipoprotein, chain D hemoglobin ypsilanti, alpha-1-antitrypsin (AAT), and beta-2-microglobulin precursor (b2M). Even if they aimed to reach a diagnostic pattern, the main limitation of the study was the low number of patients recruited [53].

Other studies have tried to use an easily accessible human sample in order to classify different grades of meningioma. In particular, Mashayekhia et al. [54] identified a low expression of TIMP1 and TIMP2 (tissue inhibitor of metalloproteinases) in serum of patients with a high grade of meningioma and compared it with controls. Examination of TIMP-1 and TIMP-2 serum levels by ELISA revealed a significant reduction of TIMP-1 and TIMP-2 concentration as compared to healthy subjects (*p* < 0.001) [54].

Despite favorable recommendations, the implementation of these procedures for the clinical preoperative and prognostic purposes has not yet achieved the expected popularity [55].

#### 4.2.4. Molecular Mechanisms of Brain-Invasive Meningioma Tumor Cells

Some studies have focused on searching for molecular biomarker expression that could correlate with BI, and this has led to controversial and not always statistically significant results. For instance, Rooprai’s group [22] refuted the correlation between secreted protein acidic and rich in cysteine (SPARC) expression and BI in different grades of meningioma, as previously proposed by Rempel et al. [56]. The use of staining techniques for glial fibrillary acidic protein (GFAP), CD44, and EMA (epithelial membrane antigen) showed to increase the sensitivity in BI detection [20,26,57].

Progress has been made in understanding the mechanisms underlying the oncogenesis in meningioma, but the pathogenetic processes implicated in BI still remain unclear. It has been documented that BI in meningioma is correlated to molecular alterations at various cellular components and in signal transmission pathways, and alterations in metalloproteases, particularly MMP-9 [26], adhesion molecules deficits, the role of the tumor microenvironment and glial cells, growth factors, and mitotic index, appear to contribute to the pathogenesis of brain infiltration [16,58,59]. For example, the invasiveness and aggressiveness of meningiomas were related to an upregulation of MMP-9 and a downregulation of E-cadherin, AKAP-12, and DEP-1 [60]. Such alterations result in increased extracellular matrix degradation and inhibition of intercellular adhesion, leading to the epithelial–mesenchymal transition phenomenon associated with the tendency of the tumor to infiltrate. In a recent paper, von Spreckelsen and colleagues [61] highlighted that transcription factors and micro RNAs (mi-RNAs) have also been shown to play a role in BI of meningiomas through post-transcriptional modification, with particular attention to miR145, Let-7d, miR-18a, and miR-200a. Interestingly, different mechanisms play a role between BI and bone invasion in some meningioma subtypes, depending on tumor location and genomic features and, among them, NF-2 and TRAF7 mutations [62].

### 4.3. Prognostic Significance and Clinical Implications

Pizem and colleagues [6] assessed, both retrospectively and prospectively, the parameters of brain infiltration in 294 meningioma specimens. They documented that BI was found more frequently in the prospective group (probably for the greater number of collected tissue blocks), in meningioma with no well-defined cleavage plane, and in those with high mitotic index (Ki-67). Conversely, they found no statistically significant association between BI and patients’ sex and age, the presence of PTBE (*p* = 0.774), tumor size, or tumor location; but interestingly, they reported a frequent association of the probability of finding BI and some histotypes (e.g., fibroblastic histotype, OR, 0.1; *p* = 0.06). However, their series lacked finding a clear association between BI, histological subtypes, and recurrence rate [6].

In a recent paper, Fioravanzo et al. [12] proposed a risk score based on five clinicopathologic features (male sex, parasagittal site, Simpson grade 3, mitotic index >6/10 HPF, and sheeting) in predicting the probability of relapse in atypical meningiomas. They analyzed 200 atypical meningiomas and showed that a score >2 was associated with a 4.7 risk of shorter disease-free survival (*p* < 0.0001). Nevertheless, in their cohort, BI alone was not associated with an increased risk of recurrence [12]. Contrary to these conclusions, in a recent bi-institutional analysis, Banan’s group [17] pointed out that BI exhibited the strongest risk of relapse (hazard ratio: 4.95) by serving as an independent predictor of progression-free interval (*p* = 0.002), clearly supporting the definition of BI as a single criterion of atypia [17]. Similar results were shared by Spille et al. [20], who emphasized BI as an independent risk factor directly associated with recurrence rate from the study of 467 patients with primary intracranial meningioma.

Regarding therapeutic approach, necrosis and BI represent strong predictors of tumor recurrence and radio-resistance, regardless of extent of resection (EOR) or adjuvant radiotherapy. Moreover, these two parameters seem to correlate with worse outcomes in PFS and OS [14]. A Kaplan–Meier analysis validated a trend for a longer mean PFI in noninvasive as compared to invasive meningioma (195 vs. 160 months, *p* = 0.065), but BI appeared to not influence OS (*p* = 0.364) [20]. Biczok et al. [3] demonstrated that BI occurs in 4–11% of cases but does not adversely affect PFS and OS in a retrospective cohort of patients with newly diagnosed WHO (2016) Grade I intracranial meningiomas.

Hence, treatment decisions (e.g., for adjuvant RT) solely based on the presence of microscopic BI should be critically analyzed.

### 4.4. Future Perspectives

As widely reported for the approach to other CNS tumors than meningioma, identifying variables and target molecules that help in predicting more aggressive behavior or not could be key elements in maximizing patients’ care. If the prognostic impact of CNS invasion in meningioma tumors becomes more established, this would be an important basis for the assessment of possible standardization for intraoperative sampling, for tissue marker assessment associated with invasive growth, and consequently for the development of innovative therapeutic approaches. In this regard, together with postsurgery irradiation therapy, specific treatment approaches targeting key molecules involved in the invasive process could be developed, leading to a personalized therapeutic choice. Extracellular matrix degradation, cell adhesion, and growth factors have been considered as potential therapy targets [2]. In the future, treatment decisions, might be based on histomorphological features, as well as molecular parameters, such as telomerase reverse transcriptase (TERT) promoter mutations, and methylation profiling, which have proven to be of prognostic value in meningiomas [63,64,65], and CSF and serum proteomics have a potential role in the early diagnosis and histopathological characterization. Further investigations and collaboration between research, oncologists, clinicians, and neurosurgeons are encouraged to provide more data and share experience in order to advance this highly interesting research area.

## 5. Conclusions

The detection of brain invasion in meningioma planning is proving to be one of the pivotal issues in neurosurgical practice, even if the prognostic and therapeutic results of its effective impact are still not homogeneous, and research is still ongoing.

Currently, the wide armamentarium is that a neurosurgeon reveals substantial different approaches, impairing the chance to have a unique point of view and a precise neuropathological diagnosis. Further studies are mandatory to assess a uniform and standardized application of BI evaluation, and the current data are promising.

## Figures and Tables

**Table 1:** Qualitative analysis of the main characteristic of the articles included in this systematic review.

Author, Year	N of Patients	Sex	Age	WHO Classification	Tumor Location	Therapy	Brain Invasion (Present)	Brain Invasion (Absent)	High Mitotic Index (×10 HPF)	Low Mitotic Index	Pre-Operative Edema	Pre-Operative MRI Analysis
McLean et al., 1993 [18]	28	15 M, 13 F	Mean 54	28 II–III	Non-skull base (not specified location)	NR	NR	NR	Present (not specified)	NR	NR	NR
McLean et al., 1993 [18]	20	not specified	Not specified	12 II; 8 III	Non-skull base (not specified location)	NR	NR	NR	NR	NR	NR	NR
Perry et al., 1998 [5]	116	63 M, 53 F	Median 60	not specified	Non-skull base (not specified location)	Surgery + RT	118	NR	> 4/10 HPF 26%; >20/10 HPF 63%	NR	NR	NR
Pizem et al., 2014 [6]	294	93 M, 201 F	Median 58	233 I; 61 II–III	146 parasagittal; 115 skull base; 33 non-skull base (not specified location)	Surgery + RT	22 (28%) benign, 33 (64%) atypical, 10 (100%) malignant	229	2.4 per 10 HPFs (mean)	NR	present	NR
Vranic et al., 2014 [7]	86	42 M, 44 F	Median 57.2	76 II; 10 III	26 falx; 36 convexity; 24 skull base	NR	25	NR	NR	NR	present	NR
Spille et al., 2016 [20]	467	136 M, 331 F	Median 57	401 I; 60 II; 6 III	66 parasagittal; 173 convexity; 221 skull base; 7 intraventricular	Surgery + RT	77% finger-like in middle skull base, 53% clustered in convexity)	NR	NR	NR	NR	NR
Adeli et al., 2018 [8]	617	176 M, 441 F	Median 59	557 I; 57 II; 3 III	215 convexity; 85 parasagittal; 271 skull base; 41 posterior fossa; 5 intraventricular	Surgery	24	593	NR	NR	554 (median)	Arachnoid layer disrupted/irregular tumor shape; calcifications; capsular contrast enhancement; heterogeneous enhancement
Hess et al., 2019 [9]	176	68 M, 108 F	Median 60	92 I; 79 II; 5 III	72 convexity, 69 skull base, 35 non-skull base (not specified location)	Surgery	38	138	NR	NR	130.7 ± 110.2 cm^3^ (volume)	Median tumor volume = 13.73 m^3^
Timme et al., 2019 [10]	2625	713 M, 1912 F	Median 61	2488 I; 137 II–III	1809 non-skull base (not specified location); 816 skull base	Surgery	136 non-skull base, 40 skull base	NR	NR	NR	NR	NR
Biczok et al., 2019 [3]	875	220 M, 655 F	Median 57	875 I	8 convexity; 400 skull base; 467 non-skull base (not specified location)	Surgery + RT	NR	NR	range 0	range 0	NR	NR
Friconnet et al., 2019 [11]	54	16 M, 38 F	Mean 58.5	41 II–III; 13 I	18 skull base; 36 non-skull base (not specified location)	Surgery	38	NR	NR	NR	26 patients	Presence of incomplete CSF rim
Fioravanzo et al., 2020 [12]	200	100 M, 100 F	Median 63	200 II	95 convexity; 63 parasagittal; 42 skull base	Surgery	94	106	82	118	NR	NR
Friconnet et al., 2020 [21]	101	34 M, 67 F	Mean 60.2	62 I; 39 II–III	36 convexity, 26 parasagittal; 5 falx; 34 skull base	NR	NR	NR	NR	NR	NR	Analysis of shape, fractal and skeleton of the tumor
Behling et al., 2020 [13]	1517	402 M, 1115 F	Median 56.8	1281 I; 232 II; 4 III	788 skull base; 574 falx; 155 not specified location	NR	Found intraoperatively 345 pt; histopathology 73 pt	intraoperative 1110; histopathological 1444	NR	NR	NR	NR
Joo et al., 2020 [14]	454	126 M, 328 F	Mean 55	397 I; 57 II–III	63 convexity; 4 falx; 16 skull base; 4 posterior fossa; 1 intraventricular; 366 non-skull base (not specified location)	NR	88	366	NR	NR	158.3 ± 114.5 (mL) volume	NR
Joo et al., 2020 [14]	150	47 M, 103 F	Mean 57.7	99 I; 48 II; 3 III	13 convexity; 6 falx; 3 skull base; 1 posterior fossa; 1 intraventricular; 126 non-skull base (not specified location)	NR	29	121	NR	NR	182.02 ± 129.43 (mL) (volume)	NR
Rooprai et al., 2020 [22]	34	13 M, 21 F	Mean 62	7 I; 26 II; 1 III	28 convexity; 6 skull base	NR	NR	NR	yes	NR	NR	NR
Park et al., 2020 [19]	131	26 M, 105 F	Mean 57.8	98 I; 29 II; 4 III	100 skull base; 31 non-skull base (not specified location)	NR	NR	NR	7.5 ± 5.7 (high grade)	1.1 ± 0.3 (low grade)	NR	Used fractal parameters
Garcia-Segura et al., 2020 [15]	181	72 M, 109 F	Mean 56.9	181 II	68 convexity; 48 falx; 65 skull base	Surgery + RT	48	133	28 patients	143 patients	NR	NR
Behling et al., 2021 [15]	1718	489 M, 1229 F	Median 70	1412 I; 285 II; 21 III	649 convexity; 893 skull base; 176 non-skull base (not specified location)	NR	108	1610	NR	NR	NR	NR
Banan et al., 2021 [17]	374	127 M, 247 F	Median 65	316 I; 58 II	75 convexity; 47 parasagittal; 174 skull base; 17 posterior fossa; 61 non-skull base (not specified location)	Surgery + RT	20	240	NR	NR	NR	NR

Abbreviations: MRI—Magnetic Resonance Imaging; NR—not recognized; RT—radiotherapy; CSF—cerebrospinal fluid.
